# Artificial Intelligence Applications in Pediatric Dentistry: A Comprehensive Review

**DOI:** 10.3390/biomimetics11050339

**Published:** 2026-05-14

**Authors:** Zeliha Hatipoğlu Palaz, Yasemin Akın, Ümmühan Karademir, Ecem Elif Çege, Mehmet Bani

**Affiliations:** 1 Department of Pediatric Dentistry, Faculty of Dentistry, Gazi University, 06490 Ankara, Turkey; yasemin.akdemir@gazi.edu.tr (Y.A.); ummuhan.karademir@gazi.edu.tr (Ü.K.); mehmetbani@gazi.edu.tr (M.B.); 2 Department of Pediatric Dentistry, Faculty of Dentistry, Karabük University, 78050 Karabük, Turkey; elifcege@karabuk.edu.tr

**Keywords:** artificial intelligence, deep learning, machine learning, pediatric dentistry

## Abstract

The application of artificial intelligence (AI) technologies in pediatric dentistry has expanded rapidly and is assuming an increasingly important role in the assessment of oral health and diagnostic processes in children. In particular, image-based analyses and data-driven predictive models offer significant potential to enhance diagnostic accuracy and reduce observer-dependent variability. This review addresses AI applications in pediatric dentistry within a structured framework, summarizing the methodological foundations of machine learning and deep learning approaches while critically evaluating the current body of evidence. It further identifies key limitations in the existing literature and outlines future research priorities, aiming to provide a perspective for the safe and sustainable clinical integration of AI in pediatric dentistry.

## 1. Introduction

Artificial intelligence (AI) is defined as a field focused on developing computational systems capable of mimicking human cognitive functions such as reasoning, learning, and problem-solving [1,2,3]. Through algorithmic approaches that simulate specific aspects of human intelligence, AI has been increasingly adopted across various medical disciplines, including dentistry [4,5].

Over the past decades, AI has been progressively integrated into dental practice, particularly in diagnostic imaging and treatment planning. Contemporary reviews emphasize that AI-based systems enhance efficiency, consistency, and accuracy in radiographic interpretation, thereby supporting optimized clinical workflows [6]. In pediatric dentistry, deep learning and image-processing algorithms have demonstrated promising performance in applications such as caries detection, anomaly identification, and developmental assessment, suggesting their potential to improve diagnostic precision and support clinical decision-making [7,8].

Among the principal subfields of AI are machine learning (ML) and deep learning (DL), both of which have gained particular prominence in medical applications [9]. Machine learning enables algorithms to learn patterns from data and generate predictive or diagnostic outputs without explicit programming. When trained on problem-specific datasets, ML models identify underlying structures and apply learned patterns to new inputs [10]. As a subset of ML, deep learning utilizes multilayer artificial neural networks to model complex relationships within data. When trained on large, high-quality datasets, DL systems achieve high levels of accuracy and are widely employed in medical image analysis and decision-support systems [11,12].

Convolutional neural networks (CNNs), a specialized form of deep learning, are particularly effective in image analysis due to their ability to automatically extract hierarchical visual features. Consequently, CNN-based architectures have assumed a central role in medical and dental imaging applications [13].

AI applications in dentistry are closely associated with the interdisciplinary principles of biomimetics, as many modern AI systems are inspired by biological neural networks and human cognitive processes. In particular, deep learning architectures such as CNNs emulate aspects of the human visual system through hierarchical feature processing, enabling automated interpretation of complex clinical and radiographic data. This biologically inspired computational approach reflects one of the core objectives of biomimetics: translating natural mechanisms into functional technological systems.

Within pediatric dentistry, AI-supported technologies further reflect biomimetic concepts through their ability to mimic human pattern recognition and adaptive analytical processes. Accordingly, the increasing integration of AI into pediatric dentistry aligns with the broader scope of biomimetics by combining biological inspiration with advanced computational methodologies to support healthcare innovation.

These technological advancements have led to substantial progress in dentistry, particularly in diagnostic imaging, risk assessment, and treatment planning. ML- and DL-based models have demonstrated high accuracy in radiographic interpretation and increasingly serve as supportive tools in clinical decision-making. In pediatric dentistry, these approaches have shown promising performance in applications such as caries detection, identification of dental anomalies, and developmental assessment, contributing to enhanced diagnostic precision and optimization of treatment planning processes [14,15].

Given the rapidly expanding body of literature in this field, a structured review of current evidence is needed to better understand the clinical applications and limitations of AI in pediatric dentistry.

The literature reviewed in this study primarily consisted of articles published within the last five years identified using the keywords “Artificial Intelligence,” “Deep Learning,” “Machine Learning,” and “Pediatric Dentistry.” Studies published in internationally indexed journals, particularly those indexed in SCIE, SSCI, and ESCI, were evaluated and narratively synthesized according to their clinical applications and methodological approaches.

The aim of this review is to comprehensively evaluate current applications of artificial intelligence in pediatric dentistry, with particular emphasis on AI- and deep learning-based methodologies and their clinical relevance.

## 2. AI Application in Pediatric Dentistry

Studies in pediatric dentistry have demonstrated that AI algorithms have been applied across a broad spectrum of clinical task [14,16]. These include plaque detection, identification and numbering of teeth on dental radiographs, detection of early childhood caries, assessment of caries risk, evaluation of the caries–pulp relationship, determination of root development stages, estimation of chronological age, detection of mesiodens and supernumerary teeth, identification of molar–incisor hypomineralization, assessment of ectopic eruption, detection of developmental anomalies such as taurodontism, categorization of fissure sealants, identification of anatomical landmarks during local anesthesia, and estimation of the dimensions of unerupted teeth.

Original artificial intelligence application studies included in this review, together with their AI models, datasets, primary clinical tasks, performance metrics, and major limitations, are systematically summarized in Table 1.

### 2.1. Oral Hygiene and Dental Plaque

Dental plaque is an organized bacterial biofilm that accumulates on tooth surfaces and plays a key role in the development of periodontal diseases [62]. Effective plaque control reduces gingival inflammation, and because persistent accumulation increases the risk of progression from gingivitis to periodontitis, mechanical plaque control is considered essential for periodontal disease prevention [63].

In the past, clinicians used explorers or disclosing solutions to identify plaque-affected areas; however, these methods were time-consuming and often uncomfortable for patients. Additionally, they have been reported to cause primarily cosmetic side effects, such as an unpleasant taste and prolonged discoloration of the lips and oral tissues [6].

In this context, the objective assessment and early detection of dental plaque and oral health status have gained increasing attention, particularly with the emergence of artificial intelligence-based approaches. You, W. et al. [17] developed a convolutional neural network (CNN) based model for dental plaque detection using 886 tooth images obtained from children aged 5–8 years, demonstrating diagnostic accuracy comparable to that of an experienced pediatric dentist (*p* > 0.05). Notably, unlike the dentist’s assessments, the model’s results remained consistent across repeated evaluations, suggesting greater reproducibility over time. However, the dataset of 886 images from a narrow age range (5–8 years) limits the generalizability of this model to broader pediatric populations with varying oral hygiene behaviors. Although performance was compared against a single pediatric dentist, the absence of multi-rater validation and external test sets constrains confidence in its clinical applicability. Nonetheless, by enabling parents and pediatric patients to visualize plaque accumulation, the model demonstrates potential for enhancing oral hygiene education and motivation—a dimension not addressed by conventional plaque indices.

Wang et al. [18] developed a machine learning-based oral health assessment tool using XGBoost and Naïve Bayesian algorithms to predict the Children’s Oral Health Status Index (COHSI) score and referral for treatment needs (RFTN) from short-form parent and child surveys. The best-performing model achieved a correlation of 0.88 with clinically determined COHSI scores, with a sensitivity of 93% and specificity of 49% on external validation data. Unlike the CNN-based image detection approach of You et al. [17], this model incorporates a broader range of clinical and demographic variables, enabling a more comprehensive assessment of oral health status. However, the low specificity indicates a considerable rate of false positives, and the reliance on parent-reported data introduces potential reporting bias, which limits its reliability for individual clinical decision-making.

Beyond clinical detection and assessment, artificial intelligence has also been explored in relation to patient-reported outcomes and quality-of-life measures associated with oral health. A study conducted to determine the impact of oral health on adolescents’ quality of life and to compare the results obtained using standard statistical methods and artificial intelligence algorithms demonstrated that both approaches yielded consistent and concordant findings [19]. In this study, the AI method employed singular value decomposition (SVD) applied to data from the Oral Impacts on Daily Performance (OIDP) scale—a validated questionnaire measuring the frequency of oral health-related difficulties in daily activities—to cluster respondents, arriving at the same groupings as the conventional t-test. The authors concluded that the integration of such AI methods into dental research and practice should be supported by dental education through the promotion of digital literacy.

Building on these patient-centered approaches, AI has also been applied to preventive oral health education through conversational tools. In a randomized study comparing a standalone chatbot (21-Day FunDee Plus) with a chatbot combined with face-to-face toothbrushing training in caregiver–child pairs (aged 6–42 months), both interventions were comparable across a 6-month follow-up in preventing caries, reducing plaque, improving dietary practices, and enhancing parental knowledge and involvement [20]. These findings suggest that AI-driven chatbots may serve as a scalable alternative to in-person instruction, particularly in settings where direct clinical access is limited. However, the authors noted that it remains unclear whether chatbot-based education alone can improve long-term caries outcomes, and called for longer evaluation periods and more robust study designs.

### 2.2. Dental Caries

In recent years, artificial intelligence (AI) and machine learning-based approaches have increasingly been applied to the early diagnosis and risk assessment of dental caries, demonstrating high diagnostic performance in radiographic and intraoral image analysis [64].

Ramos-Gomez et al. [21] employed a machine learning-based approach using a Random Forest algorithm to identify the most informative parent-reported questionnaire items for predicting the presence of active caries. They reported that this method showed good performance in the identification and classification of early childhood caries, with particular potential for screening and risk assessment in settings where face-to-face clinical examination is not feasible.

An automated machine learning (AutoML) algorithm was developed to detect early childhood caries (ECC) using a large-scale dataset comprising clinical, demographic, behavioral, and parent-reported oral health data from 6404 children aged 3–5 years, of whom 54% were identified as having ECC [22]. This approach presents a novel strategy for the early detection and prevention of ECC, and the machine learning-based model demonstrates considerable potential to enhance pediatric dental practice and improve oral health outcomes in children. The substantially larger dataset in this study [22], compared to the questionnaire-based approach of Ramos-Gomez et al. [21], conferred a notable advantage in model training and class balance.

In parallel, recent studies have emphasized that integrating biomarker data with demographic and clinical variables can further improve the discriminative ability of machine learning-based diagnostic models for ECC detection [23]. In particular, the inclusion of salivary-derived biomarkers has been shown to contribute to the differentiation between caries-affected and caries-free individuals, suggesting that such multivariable analytical frameworks may serve as supportive tools in clinical screening processes. However, the requirement for laboratory-derived salivary biomarkers substantially restricts the practical scalability of this approach in routine pediatric dental settings, where such resources are not routinely available.

Extending this multivariable perspective, dental caries risk prediction models that jointly consider environmental and genetic factors have been reported to enable the identification of high-risk individuals at the community level and to contribute to the planning of preventive oral health programs [24]. These findings highlight the importance of incorporating both biological and contextual determinants into predictive frameworks for more precise risk stratification.

Consistent with these observations, a study conducted in Korea developed prediction models for early childhood caries (ECC) using oral health data from 4195 children aged 1–5 years and demonstrated that both traditional logistic regression and machine learning-based models exhibited satisfactory performance [25]. The authors reported that these models could be effectively used for ECC prediction, identification of high-risk groups, and the implementation of active preventive interventions.

Portella et al. [26] similarly evaluated a CNN-based algorithm for detecting sound teeth and early carious lesions on occlusal surfaces. Using 2481 posterior teeth classified according to ICDAS criteria, the VGG-19 architecture demonstrated satisfactory performance in identifying early lesions and was found to be applicable as an adjunctive diagnostic tool. These findings suggest that more complex models do not always outperform simpler ones, and that model selection should reflect the clinical setting and available resources.

Beyond cross-sectional detection, machine learning-based approaches have also been used to predict the long-term development of dental caries. In a study conducted in Brazil, developed prediction models based on demographic, behavioral, and clinical variables collected during early childhood to estimate caries development in both primary and permanent teeth. The results indicated that machine learning methods may serve as effective tools for predicting caries progression using easily obtainable early childhood data [27]. Notably, the 10-year prospective design and the use of multiple algorithms—including decision tree, random forest, and XGBoost—enabled evaluation of caries trajectories over time, which is a considerable advantage over the cross-sectional approaches seen in most studies in this field.

More recently, Fadilah et al. [28] evaluated the performance of the YOLO-v8x artificial intelligence model implemented in the HI Bogi application for dental caries detection based on the ICDAS criteria. The model achieved sensitivity and specificity values exceeding 80% and demonstrated diagnostic performance comparable to that of dentists. The authors also reported that AI-assisted assessment reduced examination time by approximately fourfold and that no significant differences were observed between the results of direct clinical examination and those obtained using the application. However, variations in class-specific precision indicated limited consistency of the model in detecting certain lesion types, highlighting the need for further improvements through the use of larger and more diverse datasets and advanced architectures, such as transformer-based models, to enhance the detection of rare and complex lesions. However, variations in class-specific precision indicated limited consistency of the model in detecting certain lesion types. When compared with the VGG-19-based model of Portella et al. [26], YOLO-v8x offers the advantage of real-time lesion localization with markedly reduced examination time; however, both models share a fundamental limitation in that neither is capable of detecting interproximal caries, as both rely on intraoral photographs and occlusal surface images rather than bitewing radiographs.

### 2.3. Endodontic Applications in Pediatric Dentistry

AI-driven approaches in endodontics have shown promising performance in analyzing root canal configurations, calculating canal length, diagnosing periapical and fracture-related pathologies, and predicting the success of retreatment procedures [65]. Building upon these applications, AI has also been explored within pediatric endodontics, where developmental variations increase diagnostic complexity.

In pediatric endodontics, accurate assessment of apical closure is essential for planning pulpal treatment in developing permanent teeth. Bostancı et al. developed a CNN-based model using pseudopanoramic images derived from CBCT to automatically evaluate apical patency in permanent first molars. In a retrospective dataset of 262 CBCT scans and 147 ROI images, the model achieved precision, recall, and F1-scores between 0.79 and 0.81, with an overall accuracy and AUC of 0.80. These results suggest that deep learning may support reliable apical patency assessment and clinical decision-making in pediatric endodontics [29]. However, it should be noted that CBCT is not part of routine imaging protocols in pediatric dentistry due to its higher radiation dose, and its use is generally reserved for complex cases—a factor that substantially limits the clinical translatability of this model.

In addition to evaluating apical patency and root development, AI-driven approaches have been extended to the diagnosis of pulpal inflammatory conditions in pediatric patients. According to the study by Ma et al. [30] an EfficientNet-based CNN was used to detect irreversible pulpitis in primary molars on periapical radiographs. In this retrospective analysis of 348 images from children aged 3–8 years, the model demonstrated high diagnostic accuracy in detecting irreversible pulpitis in primary molars on periapical radiographs. These findings suggest that CNN-based approaches may enable early prediction of irreversible pulpitis by detecting subtle grayscale changes imperceptible to the human eye and may support clinical decision-making.

Beyond apical and pulpal assessment, AI-based systems have also been applied to support treatment-oriented diagnostic classification in pediatric endodontics. A recent study aimed to develop a deep learning-based clinical decision-support system that classifies furcation lesions in primary molars on panoramic radiographs and links them to corresponding treatment recommendations. A total of 387 panoramic radiographs from children aged 3–13 years were used to train and compare three object detection models. Among the tested models, RT-DETR-X demonstrated the highest diagnostic performance. Both systems show promise in supporting diagnostic and treatment planning decisions, particularly for less experienced clinicians and in resource-limited settings [31]; however, prospective clinical validation is still needed before routine implementation.

### 2.4. Deciduous and Young Permanent Tooth Detection

Precise interpretation of dental radiographs is fundamental to accurate diagnosis and effective treatment planning in clinical dentistry. The ability to correctly identify and distinguish primary and permanent teeth, as well as associated fillings and restorations, holds particular clinical significance during mixed dentition periods, where radiographic interpretation is inherently more complex due to overlapping structures, varying tooth sizes, and different stages of eruption.

In a 2024 study, Bumann et al. [32] developed a novel collaborative learning model based on the Mask R-CNN instance segmentation framework, designed to simultaneously detect and differentiate primary and permanent teeth while identifying dental fillings on panoramic radiographs. Two dedicated classifiers—one for tooth segmentation, one for filling detection—were trained on the UFBA-UESC dataset (368 radiographs) supplemented by 80 institutional images, and their outputs were aggregated through a collaborative inference mechanism. The tooth segmentation model achieved an mAP of 95.32% and F1 of 92.50%, the filling segmentation model reached an mAP of 91.53% and F1 of 91.00%, and the collaborative model further improved performance to an mAP of 94.09% and F1 of 93.41%. To the best of the authors’ knowledge, this was the first model to detect fillings across both dentition types simultaneously. However, the relatively small and geographically restricted dataset, combined with the absence of prospective validation, limits the clinical generalizability of these findings.

In a more narrowly defined task in 2021, Kılıç et al. [33] evaluated the CranioCatch AI system—based on the Faster R-CNN Inception v2 (COCO) architecture—for the automated detection and numbering of primary teeth on panoramic radiographs of 421 pediatric patients aged 5–7 years. The dataset was divided into training (*n* = 329, 6430 labels), validation (n = 46, 713 labels), and test (n = 46, 856 labels) groups, with annotations performed by an experienced pediatric dentistry using the FDI numbering system. In the test cohort, the model correctly identified 804 of 856 labeled teeth, yielding a sensitivity of 0.9804, precision of 0.9571, and F1 score of 0.9686. To the best of the authors’ knowledge, this represented the first AI system capable of individually detecting and numbering each primary tooth on pediatric panoramic radiographs. A key methodological strength of this study is its narrowly defined age range (5–7 years) and exclusive focus on the primary dentition, which reduces morphological variability and provides a well-controlled evaluation environment. However, the single-center design, limited test set size (n = 46), and restriction to fully erupted primary teeth with no permanent tooth components reduce extent of clinical applicability.

In a 2022 study, Kaya et al. [34] proposed a YOLOv4-based approach—a one-stage object detection architecture chosen for its speed and accuracy advantages over two-stage detectors such as Faster R-CNN—trained on 4545 panoramic radiographs of pediatric patients aged 5–13 years. The model performed well overall (mAP: 92.22%, F1: 0.91), but per-tooth analysis exposed a clinically relevant performance gap: anterior mandibular primary teeth were detected with considerably lower reliability (F1: 0.67) compared to posterior and maxillary teeth (F1: up to 0.97), highlighting the persistent challenge of superimposition artifacts in pediatric panoramic imaging. The substantially larger dataset used in this study, compared to Bumann et al. [32] and Kılıç et al. [33], contributed to greater model robustness across dentition types. However, the irregular class distribution across the 48 tooth classes spanning both primary and permanent dentitions and the exclusive reliance on single-institution data remain limitations, and the authors themselves note that the distributional imbalance, while potentially reflective of real-world variation, was a challenging factor during model training.

In a subsequent 2023 study, Kaya et al. [35] extended CNN-based analysis to a broader diagnostic scope using 4821 anonymized panoramic radiographs of children aged 5–13 years, applying the same YOLOv4 architecture to detect not only primary teeth and permanent tooth germs but also dental treatments including fillings, root canal treatments, and orthodontic brackets. The model demonstrated strong performance for primary tooth detection (F1: 0.95) and permanent tooth germ identification (F1: 0.90), supporting its potential utility in early detection of developmental anomalies. However, performance was markedly lower for treatment-related structures, with bracket detection yielding an F1 of 0.76, while fillings and root canal treatments showed further performance degradation—findings the authors attributed to the higher morphological variability and lower radiographic contrast associated with these restorative categories. Importantly, this study represents a methodological expansion relative to [34] in that it incorporates both structural detection and treatment classification within a single pediatric-focused framework; however, the absence of disaggregated metrics for fillings and root canal treatments in the published abstract limits a comprehensive quantitative comparison.

Taken together, these four studies reflect a rapidly maturing but still methodologically heterogeneous body of evidence in automated pediatric tooth detection. Dataset size varied considerably across studies—from 421 images in Kılıç et al. [33] to over 4800 in Kaya et al. [35]—and architectural approaches ranged from two-stage detectors (Mask R-CNN [32], Faster R-CNN [33]) to the faster one-stage YOLO framework [34,35]. Despite strong headline performance metrics across all studies, several cross-cutting limitations persist: all studies rely on retrospective, single-center datasets; none report inter-rater reliability between the AI model and clinicians of varying experience levels; and performance stratification by dentition stage or patient age subgroup is largely absent.

### 2.5. Age Estimation and Root Development Assessment

Dental development is a complex process that begins prenatally and continues until early adulthood, and its evaluation is essential for growth assessment, forensic applications, and age estimation. Beyond age determination, accurate assessment of apical status and root development plays a crucial role in endodontic treatment planning and prognosis. The presence of an open or closed apex directly influences therapeutic decisions, while variations in apical foramen diameter may affect pulpal healing outcomes. Although panoramic radiographs are widely used for this purpose, their subjective interpretation may introduce variability, underscoring the need for objective and reliable evaluation methods [39,40].

In a 2022 study, Lee et al. [36] examined the relationship between 18 radiomorphometric parameters from panoramic radiographs and chronological age using five machine learning algorithms in 471 patients aged 11–69 years. Patients were grouped into three broad and six narrower age categories. In the three-group classification, AUC values were higher for the young (0.85–0.87) and old groups (0.79–0.88), while the adult group showed lower performance (~0.73), a pattern consistent across the six-group analysis. Feature analysis indicated that pulp area parameters were most informative for younger individuals, whereas age-related damage features (e.g., restorations, implants, periodontitis) were more predictive in older groups. No significant difference was observed between linear and nonlinear models (*p* > 0.05), suggesting a largely linear structure of age-related radiographic information. However, the study’s focus on permanent dentition in a Korean population and the imbalance toward younger individuals limit its applicability to pediatric age estimation.

In another significant study on chronological age estimation, Zaborowicz et al. (2022) developed three separate deep neural network models using panoramic radiographs of 619 children and adolescents aged 4–15 years [37]. Conducted at Poznan University in Poland, the researchers developed 21 original indicators based on dental and bone parameters, and trained three distinct models via the H2O.ai platform: a combined male-female model, a female-only model, and a male-only model. Examination of the performance metrics reveals that the highest accuracy was achieved by the male-specific model, with an MAE of 2.34 months, RMSE of 5.58 months, and R^2^ = 0.957. The combined model demonstrated lower accuracy, with an MAE of 4.61 months and R^2^ = 0.932. Notably, R^2^ values in the validation set were considerably lower than those in the training set, suggesting a risk of overfitting given the limited sample size. The primary methodological limitation is that manual measurement of indicators using ImageJ software version 1.52a may introduce inter-observer variability, and the approach does not constitute a truly end-to-end automated workflow.

Beyond directly linking teeth to chronological age, deep learning approaches have also been applied to the automatic classification of tooth developmental stages. Dong et al. (2023) developed a two-stage system for Demirjian staging of the full permanent dentition using panoramic radiographs of 673 children aged 3–14 years [38]. In the first stage, a YOLOv3-based model achieved tooth localization with AP_50_ = 97.50% and mIOU = 0.8078; in the second stage, SOS-Net, which leverages bilateral facial symmetry and the ordinal relationships among stages, attained W_F1 = 79.04% and AUC > 0.94 across all Demirjian stages. In the age estimation task, an MAE of 0.690 years was achieved, outperforming the majority of compared methods.

In 2024, Kurt et al. [39] evaluated YOLOv5 for automated detection and classification of Demirjian developmental stages in 1458 pediatric patients aged 5–14 years. The dataset was divided into training, validation, and test sets, with annotations performed by multiple clinicians. In the test cohort, the model achieved a sensitivity of 0.99, precision of 0.72, and an F1 score of 0.84, indicating excellent detection capability but a tendency toward false positives. This imbalance was attributed to class distribution challenges and variability in multi-rater labeling. Notably, this study represents one of the first CNN-based approaches targeting Demirjian-stage classification in pediatric panoramic radiographs. However, its single-center design, class imbalance across developmental stages, and lack of external validation limit generalizability.

In a more clinically targeted application, Kayaci et al. [40] developed a CNN-based framework for detecting and classifying root development stages of permanent first molars on pediatric panoramic radiographs. Using 1629 images from 409 patients aged 5–14 years, labeled according to the five-stage Cvek classification, YOLOv7 achieved high detection performance (precision 98.4%, recall 97.6%, mAP 99.3%). For stage classification, VGG-19 outperformed other models, yielding an overall accuracy of 64.21%, with higher performance in mandibular than maxillary teeth, likely due to anatomical superimposition in the maxilla. Clinically relevant binary classification (open vs. closed apex) achieved 84.57% accuracy, improving to 94.89% when ambiguous stages were excluded, highlighting the difficulty of distinguishing late-stage categories on two-dimensional images. However, the absence of clinician comparison, single-center data, and lack of ROC/AUC metrics limit the generalizability of the findings.

Taken together, these four studies reflect both the rapid progress and the persistent limitations of deep learning-based dental age and stage assessment. Across all architectures, object detection models consistently achieved high tooth localization performance—YOLOv3 attained AP_50_ = 97.50% in Dong et al., while YOLOv5 and YOLOv7 yielded F1 = 0.84 and mAP = 99.3% in Kurt et al. and Kayaci et al., respectively—suggesting that tooth detection is now a largely solved problem. The more meaningful performance differences emerge at the classification stage: Dong et al.’s SOS-Net achieved W_F1 = 79.04% across nine Demirjian stages, whereas Kayaci et al.’s VGG-19 reached only 64.21% for a five-stage root classification task, likely reflecting both the greater morphological complexity of full-dentition staging and differences in sample size and label quality. For direct age estimation, Zaborowicz et al. reported errors as low as 2.34 months (MAE) using hand-crafted indicators, while Dong et al. achieved 0.690 years through fully automated staging—a methodologically important distinction, as the former relies on manual softJ measurements that introduce inter-observer variability. Critically, all four studies share the same fundamental limitation: single-center datasets with no external validation, raising unresolved questions about generalizability across ethnic populations, imaging protocols, and clinical settings.

### 2.6. Supernumerary Teeth

In pediatric dentistry, artificial intelligence (AI)-based approaches have increasingly been applied to the detection and assessment of supernumerary teeth, particularly during the primary and mixed dentition periods when early diagnosis is critical [2,47]. Supernumerary teeth, most commonly mesiodens, may remain clinically undetected in early childhood yet lead to eruption disturbances, impaction of permanent incisors, and orthodontic complications if diagnosis is delayed [66].

Ahn et al. (2021) evaluated four CNN architectures for mesiodens classification on 1100 panoramic radiographs using five-fold cross-validation [41]. ResNet-101 and Inception-ResNet-V2 achieved the highest performance (accuracy: 0.927 and 0.924; AUC: 0.941 and 0.932; F1-score: 0.928 and 0.925), while SqueezeNet showed the weakest results (accuracy: 0.833; AUC: 0.862), consistent with the positive relationship between network depth and classification accuracy. Class activation map analysis confirmed that models focused on anatomically relevant regions. Although all deep learning models outperformed general dentists in speed (∼1.5 s vs. 812 s for 100 images), their accuracy remained below that of experienced pediatric specialists (accuracy: 0.99) [41].

Similarly, Ha et al. (2021) developed a YOLOv3-based detection model trained on 612 panoramic radiographs across all dentition stages, achieving internal accuracy of 96.2% (sensitivity: 95.4%; specificity: 96.9%) and external accuracy of 89.8%, with mixed dentition yielding the highest performance [42]. Mine et al. (2022) reported more modest results in a pilot study restricted to early mixed dentition (n = 220), where VGG16-TL achieved accuracy of 84.0%, sensitivity of 85.0%, and AUC of 0.87—lower figures likely reflecting the greater anatomical complexity of transitional dentition [2].

Kim et al. (2022) introduced a fully automated two-step pipeline combining DeepLabV3+ segmentation with Inception-ResNet-V2 classification on 988 panoramic radiographs, eliminating the need for manual ROI selection [43]. Classification using the automatically segmented ROI achieved accuracy, F1-score, and AUC of 0.971—significantly outperforming manually segmented ROI-based results (AUC: 0.892; *p* < 0.05). Jeon et al. (2022) extended this work to periapical radiographs (n = 600), comparing YOLOv3 (accuracy: 97.5%; sensitivity: 100%), RetinaNet (98.3%; sensitivity: 100%), and EfficientDet-D3 (99.2%; specificity: 100%) [44]. The trade-off between sensitivity and specificity across algorithms is clinically meaningful: higher sensitivity minimizes missed diagnoses, while higher specificity reduces unnecessary referrals. Okazaki et al. (2022) demonstrated that multiclass classification of supernumerary teeth and odontomas simultaneously is feasible but substantially more challenging, with macro-average accuracy dropping to 70.0% compared to 83.3% for binary tasks [45]. Uzel et al. (2025) recently applied YOLOv8 to 2000 pediatric panoramic radiographs and reported perfect classification performance (accuracy, precision, recall, F1-score: 100%), but the segmentation model showed high precision (1.00) with markedly low recall (0.38) and F1-score (0.55), revealing the persistent gap between classification and precise lesion localization [47]. Mladenovic et al. (2023) further illustrated that commercially available AI segmentation tools vary substantially in their ability to detect multiple supernumerary teeth, reinforcing the need for clinical oversight [46].

Across studies, binary classification on panoramic radiographs consistently yields accuracy values of 84–97%, whereas performance declines when tasks shift toward spatial localization or multiclass discrimination. Common limitations include single-institution datasets, limited external validation, and the absence of standardized performance reporting, which hinder direct cross-study comparisons. Future research should prioritize multicenter validation and models capable of simultaneous detection, localization, and characterization of supernumerary teeth across all dentition stages.

### 2.7. Ectopic Eruption

Ectopic eruption is a developmental disturbance characterized by the eruption of permanent teeth along an abnormal path, most commonly observed during the mixed dentition period. Panoramic radiography is the primary imaging modality for early detection of ectopic eruption; however, diagnostic accuracy may be limited by anatomical complexity specific to mixed dentition and by inter-observer variability [67]. In this context, artificial intelligence (AI)-based image analysis systems have been increasingly investigated as supportive tools to improve the early and consistent identification of ectopic eruption in pediatric populations [48,50].

In 2022, Liu et al. developed a deep learning-based semi-automated screening system for detecting ectopic eruption of maxillary permanent first molars using 1580 panoramic radiographs from children aged 4–9 years. The fusion model achieved a sensitivity of 0.89, specificity of 0.86, positive predictive value of 0.86, and F1-score of 0.877, with AUC values of 0.944–0.946. AI-assisted evaluation outperformed pediatric dentists (mean F1-score: 0.857) and further improved their performance when used as a decision-support tool (F1-score: 0.921), suggesting that such systems may enhance diagnostic consistency and facilitate detection of early-stage cases that might otherwise be overlooked [48].

Similarly, Zhu et al. (2022) proposed an nnU-Net-based deep learning model for the automated detection and segmentation of ectopically erupting maxillary first permanent molars in children during early mixed dentition. Using 285 panoramic radiographs with 438 ectopic molar regions, the model achieved an IoU of 0.834, a precision of 0.845, an F1-score of 0.902, and an overall accuracy of 0.990. When compared with dentists of varying levels of clinical experience, the AI model demonstrated significantly greater diagnostic consistency and accuracy. These findings underscore the potential of AI-based systems in screening low-prevalence but clinically significant eruption anomalies [49].

Adopting a more comprehensive framework, Yu et al. (2025) developed a multi-stage deep learning system integrating tooth segmentation, developmental stage classification, and ectopic eruption detection using 1576 panoramic radiographs from children aged 6–12 years. For tooth segmentation, the model achieved an IoU of 0.959, a precision of 0.993, a sensitivity of 0.966, and an F1-score of 0.979. For ectopic eruption detection specifically, the AI model achieved an IoU of 0.798, a precision of 0.906, a specificity of 0.910, and an F1-score of 0.888—comparable to or exceeding the performance of two of the three dentists evaluated (Dentist 2 F1: 0.857; Dentist 3 F1: 0.881), and significantly surpassing Dentist 1 (F1: 0.812; McNemar *p* < 0.05). Compared with earlier models that treated ectopic eruption primarily as an isolated radiographic finding, the multi-stage design used by Yu et al. incorporated developmental context, which may explain its robust diagnostic performance and closer agreement with experienced clinicians.

Taken together, these studies show that AI-based systems can achieve moderate-to-high diagnostic performance in ectopic eruption detection, but their clinical utility depends strongly on task design. Screening models, such as that of Liu et al., may be valuable for early identification and clinician support, whereas segmentation-based approaches, such as Zhu et al.’s nnU-Net model, provide more precise localization. Multi-stage systems, such as Yu et al.’s framework, appear more clinically comprehensive because they integrate tooth development and eruption status within a single diagnostic pipeline. Nevertheless, the available evidence remains limited by retrospective study designs, restricted datasets, limited external validation, and potential class imbalance due to the relatively low prevalence of ectopic eruption. Future studies should prioritize multicenter validation, standardized annotation protocols, and evaluation of AI-assisted diagnosis in real-world pediatric dental workflows.

### 2.8. Impacted Teeth

Impacted teeth are a clinically important developmental anomaly in pediatric dentistry, most frequently involving maxillary canines during the mixed dentition period. Delayed eruption may lead to root resorption of adjacent teeth and complex orthodontic complications [68]. Although panoramic radiography is commonly used for early detection, conventional assessment depends on time-consuming measurements and is prone to interobserver variability [52]. Consequently, AI-based image analysis has been explored as a method to enhance the accuracy and consistency of impacted tooth detection and classification in pediatric patients [51].

Aljabri et al. (2022) applied transfer learning using four CNN architectures to classify maxillary canine impaction type from panoramic radiographs of 268 balanced cases. Among the tested models, Inception V3 achieved the highest performance, with an accuracy of 0.926, a precision of 0.936, a recall of 0.936, and an F1-score of 0.936, while DenseNet-121 and VGG-16 showed substantially lower performance (accuracy: 0.685 and 0.574, respectively), highlighting the sensitivity of classification outcomes to both model architecture and data balancing strategy [51].

Zhang et al. (2025) integrated a deep learning-based automatic landmark detection system with three established logistic regression models for maxillary canine impaction prediction on 102 panoramic radiographs from patients aged 7–14 years. The Margot et al. model augmented with AI-assisted landmark localization achieved the highest performance, with a sensitivity of 95%, a specificity of 86%, an accuracy of 91%, and an AUC of 0.97, while the Alqerban model performed poorly (AUC: 0.20), likely attributable to its limited original sample size and multicollinearity among predictors. This study highlighted that AI-assisted landmark detection not only reduces manual measurement burden but also enhances the interpretability of prediction models—a key advantage over opaque end-to-end classification systems [52].

Moving beyond classification and landmark prediction, Kim et al. (2024) applied a U-Net-based semantic segmentation model with ResNet encoders to detect impacted mesiodens on 850 pediatric panoramic radiographs. The best-performing configuration achieved a Jaccard index of 0.924, a Dice coefficient of 0.938, and an F1-score of 0.95—comparable to general dental practitioners (F1: 0.96)—while completing diagnosis in approximately 7.5 s versus over 600 s for human experts (*p* < 0.05) [53].

Collectively, these studies demonstrate that AI applications for impacted teeth are evolving from simple image classification toward more clinically interpretable and anatomically detailed systems. CNN-based classification models generally achieve high performance in balanced datasets; however, landmark-assisted and segmentation-based approaches may offer greater clinical relevance by improving visualization of anatomical relationships and supporting treatment planning decisions. Importantly, methodological differences among studies—including variations in imaging modality, annotation strategy, and task definition—substantially influence reported performance metrics, making direct comparisons challenging. Despite encouraging results, the current evidence remains limited by retrospective single-center datasets, small sample sizes, lack of external validation, and limited integration of three-dimensional imaging data. Future research should therefore prioritize multicenter prospective validation studies and clinically explainable AI systems that can be integrated into orthodontic and pediatric dental workflows.

### 2.9. Molar–Incisor Hypomineralization (MIH) and Enamel Defects

Molar–incisor hypomineralization (MIH) and other developmental enamel defects are common yet clinically challenging conditions in pediatric dentistry. Clinical diagnosis relies heavily on visual inspection and clinician experience, mild cases are often underdiagnosed [69]. Consequently, artificial intelligence (AI)-based image analysis has been explored to improve diagnostic objectivity and consistency [70,71].

Several studies have reported that CNN-based models developed for MIH detection using clinical photographs achieve accuracy rates exceeding 80–85% and demonstrate performance comparable to experienced clinicians in differentiating MIH from fluorosis and hypoplasia. These systems have also been shown to reduce interobserver variability and improve the detection of mild lesions [70,71,72,73].

Beyond detection-focused approaches, Jaiswal et al. (2024) introduced an AI-driven computerized system to quantify the percentage of hypomineralized opacity using 50 standardized images. The model reported lesion values ranging from 7.29% to 71.21% (mean: 34.51%), with validation results between 10.29% and 67.27% (mean: 35.32%), showing no statistically significant difference (*p* = 0.7186). This suggests that AI can provide objective and reproducible quantification of lesion extent, addressing a key limitation of prior studies that primarily focused on detection rather than severity assessment [54].

Taken together, AI-based approaches demonstrate high performance in MIH detection; however, variability in segmentation accuracy and dataset dependency remains. Importantly, emerging quantification-based models extend beyond detection by enabling standardized assessment of lesion severity, which may improve clinical decision-making and treatment planning.

### 2.10. White Spot Lesions

White spot lesions (WSLs) are early enamel demineralization areas characterized by an intact surface layer. Diagnosis based solely on visual inspection may be challenging, especially in mild cases, and is dependent on clinician experience. Therefore, artificial intelligence (AI)-based image analysis methods have been investigated to support standardized and objective detection of WSLs [55,56].

Early deep learning approaches primarily focused on classification-based diagnosis. Askar et al. (2021) developed a SqueezeNet-based model for WSL detection and reported accuracy values ranging from 0.81 to 0.84, with relatively high specificity (0.85–0.86) but lower sensitivity (0.58–0.66). These findings indicate that the model was more successful in identifying healthy structures than subtle demineralization lesions, suggesting a tendency toward false-negative classifications. Nevertheless, the study demonstrated the feasibility of AI-assisted WSL assessment using relatively lightweight CNN architectures [55].

More recent approaches have focused on improving detection performance. Chung et al. (2025) developed a TW-YOLO model that demonstrated improved agreement (κ = 0.76) and higher detection performance compared to conventional YOLOv5, likely due to better preservation of high-resolution image features and improved identification of small lesions [57]. However, performance appears to be influenced by dataset size. Ozsunkar et al. (2024) reported lower performance using a YOLOv5x model (precision: 0.786, recall: 0.618, AUC: 0.712), highlighting the feasibility of automated WSL detection despite moderate sensitivity [56]. Methodological differences further contribute to variability. Askar et al. (2021) focused on classification of lesion presence, whereas Chung et al. (2025) applied an object detection approach to localize lesions, which may explain differences in reported performance metrics [56,57].

More recent studies have increasingly focused on object detection and lesion localization rather than simple binary classification. Ozsunkar et al. (2024) evaluated a YOLOv5x-based model and reported a precision of 0.786, recall of 0.618, and AUC of 0.712. Although these findings confirmed the potential utility of automated WSL detection, the relatively low recall values suggested persistent difficulty in identifying smaller or less distinct lesions [56]. In contrast, Chung et al. (2025) developed a TW-YOLO model that demonstrated improved agreement (κ = 0.76) and higher detection performance compared with conventional YOLOv5 architectures. The improved performance may be attributable not only to architectural refinements but also to better preservation of high-resolution lesion features, which is particularly important for detecting small demineralization areas and lesion boundaries [57]. Differences in reported performance across studies appear to be strongly influenced by methodological design and task definition. Classification-based models generally demonstrate more stable and higher performance metrics because they focus on determining lesion presence rather than precise localization. In contrast, object detection systems require accurate spatial identification of lesion boundaries, making them more sensitive to image quality, lesion size, and dataset variability. Consequently, detection-based approaches may show greater performance fluctuation despite providing more clinically relevant spatial information.

Taken together, AI-based models can achieve moderate to high accuracy in WSL detection; however, performance varies depending on task definition, dataset size, and the ability to detect small lesions. Classification approaches appear more consistent, whereas detection-based methods show greater variability but provide more clinically relevant spatial information.

### 2.11. Classification of Dental Trauma

Early diagnosis and appropriate treatment of traumatic dental injuries are essential for preventing post-traumatic complications and improving the prognosis of affected teeth and surrounding tissues [74]. Given the critical role of early intervention, rapid and accurate diagnostic approaches are of paramount importance. In addition to conventional clinical and radiographic methods, artificial intelligence (AI)-based systems, particularly deep learning models, have emerged as promising tools to support and accelerate the diagnostic process [58,59].

Recent studies have increasingly focused on the diagnostic performance of deep learning models in the radiographic assessment of dental trauma. In 2025, Bani-Hani et al. [58] trained a CNN model on 72 periapical radiographs (108 fractures) to classify tooth fractures into four categories. The model achieved an overall accuracy of 78.7%, while binary classification performance reached up to 99.1%, highlighting its potential in simplified diagnostic scenarios.

In the same year, Sarıoğlu et al. [59] evaluated three YOLO-based models (v8, v11, v12) using 1374 panoramic radiographs from pediatric patients. The models showed consistently higher performance in detecting hard tissue injuries compared to periodontal injuries, with AUC values ranging from 0.69–0.73 and 0.61–0.69, respectively. Among them, YOLOv12 demonstrated the best performance (F1-score: 0.762), although overall accuracy remained moderate.

The performance differences between these studies largely reflect the inherent limitations of the imaging modalities used. While periapical radiographs provide high-detail, localized views with minimal superimposition—thereby enhancing model performance—panoramic radiographs introduce greater anatomical overlap, reducing detection accuracy, particularly for periodontal injuries. Additionally, the lower performance in detecting conditions such as luxation may be explained by radiographic similarities with mixed dentition patterns and dental crowding, as well as the absence of clinical context in model training.

Despite promising results, common limitations—including single-center datasets and lack of multimodal integration—highlight the need for future AI systems that combine radiographic data with clinical findings and patient history to improve diagnostic reliability and clinical applicability [58,59].

### 2.12. Fissure Sealant Categorization

Pit and fissure sealants are preventive materials applied to the occlusal surfaces of primary and permanent molars, and their clinical success largely depends on retention. Loss of retention directly influences clinical decision-making, often requiring re-application [75].

In a 2021 study, Schlickenrieder et al. evaluated a CNN-based model for automated detection and classification of fissure sealants using 2352 high-resolution intraoral photographs. Sealants were categorized into four groups (unsealed, intact, sufficient, and insufficient) to enable early detection of material deterioration and support timely clinical intervention. The ResNeXt-101-32x8d model achieved high detection performance (accuracy: 98.7%, AUC: 0.996), with overall classification accuracy of approximately 90%. Category-specific performance varied, with accuracies of 89.6% (intact), 83.2% (sufficient), and 92.4% (insufficient) [60].

These findings suggest that deep learning models can reliably support sealant evaluation in clinical settings. However, the relatively lower performance in intermediate categories highlights the challenge of distinguishing subtle differences in retention status, which may limit decision-making in borderline cases.

### 2.13. AI in Behavioral Management

Behavior management in pediatric dentistry plays a fundamental role in facilitating children’s safe and effective adaptation to dental treatment. In a 2024 mini-review, Acharya et al. [61] examined the role of AI-based tools in this domain, identifying virtual reality (VR), real-time emotion recognition systems, and gamification-based digital applications as the principal intervention modalities. With respect to VR, the authors cited evidence from a randomized controlled trial in which children exposed to an immersive VR environment prior to a procedure demonstrated significantly lower anxiety levels and improved behavioral cooperation compared to controls. AI-driven emotion recognition systems were similarly described as tools capable of monitoring children’s fear responses in real time and enabling clinicians to individualize distraction strategies accordingly. Gamification-based applications were discussed as mechanisms to enhance motivation and encourage sustained positive behavioral change, with review-level evidence indicating a significant association between gamification and health-oriented behavioral modifications [14]. However, Acharya et al. explicitly acknowledged key limitations, including the risk of misinterpreting children’s nonverbal cues, potential for unfamiliar AI tools to inadvertently heighten anxiety, and pronounced data privacy concerns specific to pediatric populations. Importantly, none of the described systems were evaluated with standardized quantitative metrics, which reflects a broader gap in the field: the absence of controlled clinical trials reporting objective outcome measures for AI-assisted behavior management in pediatric dentistry.

## 3. Conclusions

Artificial intelligence has rapidly expanded across multiple domains of pediatric dentistry, demonstrating promising performance in diagnostic imaging, risk prediction, developmental assessment, anomaly detection, endodontic evaluation, trauma classification, preventive care monitoring, and behavior management. Across the reviewed studies, AI- and deep learning-based systems have consistently shown high diagnostic accuracy, improved consistency, and enhanced efficiency, particularly in image-based analyses of panoramic and intraoral data. These technologies appear to reduce observer-dependent variability, support early detection of clinically significant conditions, and facilitate more standardized clinical decision-making.

Despite these encouraging findings, AI systems should currently be regarded as adjunctive tools rather than replacements for clinical expertise. Variability in datasets, model architectures, and validation strategies underscores the need for larger multicenter studies, external validation, and integration into real-world clinical workflows. When combined with professional judgment and ethical implementation, AI has strong potential to enhance precision, efficiency, and patient-centered care in pediatric dentistry.

## 4. Future Directions

Future research should focus on developing large, diverse, and multicenter datasets to improve the generalizability and robustness of AI models. Prospective and real-world clinical studies are needed to validate current findings and assess the impact of AI on clinical decision-making and treatment outcomes. Standardization of methodologies, including data annotation and performance metrics, is also essential to enable meaningful comparisons across studies.

In addition, integrating multimodal data (clinical, radiographic, and biological) may enhance diagnostic and predictive accuracy. Ethical considerations, data privacy, and regulatory frameworks should be addressed to ensure safe implementation. Finally, incorporating AI into dental education will be crucial for improving clinicians’ digital competence and facilitating its effective use in pediatric dentistry.

## Figures and Tables

**Table 1:** Artificial Intelligence Application Studies in Pediatric Dentistry.

Clinical Area	Study	AI Model/ Architecture	Dataset	Main Task	Key Findings/ Performance	Main Limitation
Oral hygiene/plaque detection	You et al. (2020) [17]	CNN	886 tooth images (5–8 years)	Dental plaque detection	Comparable to pediatric dentist; reproducible results	Small dataset; narrow age range
Oral health assessment	Wang et al. (2020) [18]	XGBoost, Naïve Bayes	Parent/child surveys	COHSI and RFTN prediction	Correlation: 0.88; sensitivity: 93%; specificity: 49%	Low specificity; reporting bias
Oral health QoL	Gajic et al. (2021) [19]	SVD-based AI clustering	OIDP questionnaire data	Quality-of-life clustering	AI findings concordant with conventional statistics	Limited direct clinical applicability
Oral health chatbot	Hunsrisakhun et al. (2025) [20]	Chatbot-based intervention	Caregiver–child pairs	Oral health education	Comparable to face-to-face instruction	Long-term outcomes unclear
ECC screening	Ramos-Gomez et al. (2021) [21]	Random Forest	Questionnaire-based data	Active caries prediction	Effective screening potential	Questionnaire dependency
ECC detection	Karhade et al. (2021) [22]	AutoML	6404 children	ECC detection	Large-scale predictive performance	Limited external validation
ECC biomarker analysis	Koopaie et al. (2021) [23]	ML-based multivariable analysis	Salivary biomarkers + clinical data	ECC discrimination	Improved differentiation of ECC status	Limited scalability
Caries risk prediction	Pang et al. (2021) [24]	Machine learning	Environmental/genetic data	Caries risk prediction	Effective high-risk identification	Population dependency
ECC prediction	Park et al. (2021) [25]	ML + logistic regression	4195 children	ECC prediction	Satisfactory predictive performance	Population-specific dataset
Early caries detection	Portella et al. (2023) [26]	VGG-19 CNN	2481 posterior teeth	Occlusal caries detection	Successful ICDAS classification	Inability to detect proximal lesions
Caries progression prediction	Toledo Reyes et al. (2023) [27]	Decision tree, RF, XGBoost	Longitudinal cohort	Caries progression prediction	Long-term predictive capability	Complex longitudinal design
Caries localization	Fadilah et al. (2025) [28]	YOLO-v8x	Intraoral photographs	ICDAS-based lesion detection	Sensitivity/specificity >80%; faster examination	Variable class-specific precision
Apical patency assessment	Bostancı et al. (2025) [29]	CNN	262 CBCT scans	Apical patency detection	Accuracy/AUC: 0.80	CBCT not routine in children
Irreversible pulpitis	Ma et al. (2025) [30]	EfficientNet CNN	348 radiographs	Pulpitis detection	High diagnostic accuracy	Retrospective design
Furcation lesion diagnosis	Karamüftüoğlu et al. (2025) [31]	RT-DETR-X	387 panoramic radiographs	Furcation lesion classification	Highest performance among tested models	No prospective validation
Mixed dentition analysis	Bumann et al. (2024) [32]	Mask R-CNN collaborative model	448 panoramic radiographs	Tooth/filling segmentation	mAP: 94.09%; F1: 93.41%	Small geographically restricted dataset
Primary tooth numbering	Kılıç et al. (2021) [33]	Faster R-CNN Inception v2	421 panoramic radiographs	Tooth detection/numbering	Sensitivity: 0.9804; F1: 0.9686	Limited test cohort
Tooth detection	Kaya et al. (2022) [34]	YOLOv4	4545 panoramic radiographs	Tooth detection	mAP: 92.22%; F1: 0.91	Lower mandibular anterior performance
Diagnostic charting	Kaya et al. (2023) [35]	YOLOv4	4821 panoramic radiographs	Tooth/treatment detection	F1: 0.95 (primary teeth)	Lower restorative detection accuracy
Age group estimation	Lee et al. (2022) [36]	Five ML algorithms	471 radiographs	Age group prediction	AUC: 0.79–0.88	Limited pediatric specificity
Chronological age estimation	Zaborowicz et al. (2022) [37]	Deep neural network	619 radiographs	Age estimation	MAE: 2.34 months	Potential overfitting
Demirjian staging	Dong et al. (2023) [38]	YOLOv3 + SOS-Net	673 radiographs	Developmental staging	W_F1: 79.04%; MAE: 0.690 years	Complex workflow
Demirjian classification	Kurt et al. (2024) [39]	YOLOv5	1458 radiographs	Developmental stage classification	Sensitivity: 0.99; F1: 0.84	Class imbalance
Root development assessment	Kayaci et al. (2025) [40]	YOLOv7 + VGG-19	1629 images	Root stage classification	mAP: 99.3%; binary accuracy: 94.89%	Limited generalizability
Supernumerary tooth detection	Mine et al. (2022) [2]	VGG16 transfer learning	220 radiographs	Supernumerary tooth detection	Accuracy: 84%; AUC: 0.87	Small pilot dataset
Mesiodens classification	Ahn et al. (2021) [41]	ResNet-101/Inception-ResNet-V2	1100 radiographs	Mesiodens classification	Accuracy: 0.927; AUC: 0.941	Lower than specialists
Mesiodens detection	Ha et al. (2021) [42]	YOLOv3	612 radiographs	Mesiodens detection	Accuracy: 96.2%	External performance reduction
Automated mesiodens diagnosis	Kim et al. (2022) [43]	DeepLabV3+ + Inception-ResNet-V2	988 radiographs	Segmentation/classification	AUC: 0.971	Complex pipeline
Impacted mesiodens detection	Jeon et al. (2022) [44]	YOLOv3, RetinaNet, EfficientDet-D3	600 radiographs	Mesiodens detection	Accuracy up to 99.2%	Algorithm-dependent variability
Dental anomaly classification	Okazaki et al. (2022) [45]	Deep learning classifier	Radiographic images	Multiclass anomaly classification	Accuracy: 70%	Multiclass complexity
Multiple supernumerary teeth	Mladenovic et al. (2023) [46]	AI segmentation systems	Multiple supernumerary teeth	Segmentation	Variable performance across systems	Requires clinician oversight
Supernumerary detection/segmentation	Uzel et al. (2025) [47]	YOLOv8	2000 radiographs	Detection and segmentation	Classification accuracy: 100%	Low segmentation recall
Ectopic eruption screening	Liu et al. (2022) [48]	Fusion deep learning model	1580 radiographs	EE screening	F1-score: 0.877; AUC: 0.944–0.946	Retrospective design
Ectopic eruption segmentation	Zhu et al. (2022) [49]	nnU-Net	285 radiographs	EE segmentation	Accuracy: 0.990; F1: 0.902	Small dataset
Ectopic eruption diagnosis	Yu et al. (2025) [50]	Multi-stage DL framework	1576 radiographs	EE detection	F1-score: 0.888	Complex workflow
Canine impaction classification	Aljabri et al. (2022) [51]	Inception V3	268 radiographs	Impaction classification	Accuracy: 0.926; F1: 0.936	Small balanced dataset
Canine impaction prediction	Zhang et al. (2025) [52]	DL landmark + regression	102 radiographs	Impaction prediction	AUC: 0.97	Limited dataset
Impacted mesiodens segmentation	Kim et al. (2024) [53]	U-Net + ResNet	850 radiographs	Mesiodens segmentation	Dice: 0.938; F1: 0.95	Single-center data
MIH quantification	Jaiswal et al. (2024) [54]	AI quantification system	50 standardized images	Lesion severity quantification	No significant validation difference	Small sample size
WSL classification	Askar et al. (2021) [55]	SqueezeNet	Clinical images	WSL classification	Accuracy: 0.81–0.84	Lower sensitivity
WSL detection	Ozsunkar et al. (2024) [56]	YOLOv5x	Clinical images	WSL detection	Precision: 0.786; AUC: 0.712	Moderate recall
WSL localization	Chung et al. (2025) [57]	TW-YOLO	Clinical images	WSL localization	κ = 0.76	Dataset dependency
Dental trauma classification	Bani-Hani et al. (2025) [58]	CNN	72 periapical radiographs	Fracture classification	Accuracy: 78.7%	Small dataset
Dental trauma segmentation	Sarıoğlu et al. (2025) [59]	YOLOv8/v11/v12	1374 radiographs	Trauma segmentation	Best F1-score: 0.762	Moderate overall accuracy
Fissure sealant classification	Schlickenrieder et al. (2021) [60]	ResNeXt-101-32x8d	2352 intraoral photographs	Sealant classification	Accuracy: 98.7%; AUC: 0.996	Borderline category difficulty
Behavioral management	Acharya et al. (2024) [61]	VR/emotion recognition/gamification	Review-level evidence	Anxiety reduction	Improved cooperation reported	Lack of standardized AI metrics

## Data Availability

No new data were created or analyzed in this study. Data sharing is not applicable.
